# Supplementary material for: Structural Insights into Neonicotinoids and N-Unsubstituted Metabolites on Human nAChRs by Molecular Docking, Dynamics Simulations, and Calcium Imaging
Source: Int J Mol Sci. 2023 Aug 24;24(17):13170. doi: 10.3390/ijms241713170 (PMC10487998; doi:10.3390/ijms241713170)
Supplement: Supplementary file 1 [file ijms-24-13170-s001.zip › ijms-2556915-SI.pdf]

Supporting information

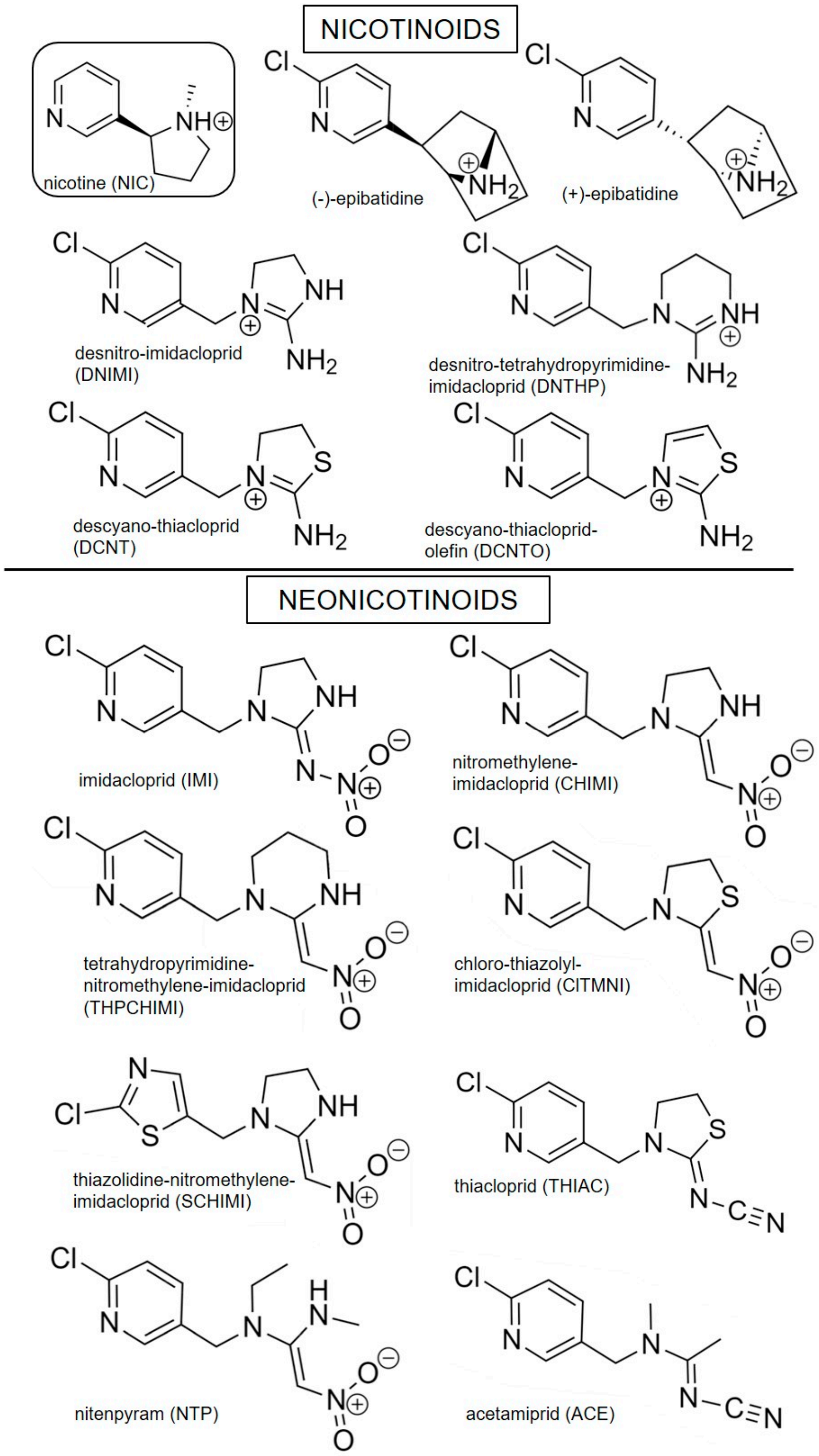

Figure S1. 2D-structures of the 15 ligands that were subjected to the ensemble docking approach. Neonicotinoids are represented by imidacloprid (IMI), thiacloprid (THIAC), nitenpyram (NTP), and acetamiprid (ACE), amongst the neonicotinoid analogues are nitromethylene-imidacloprid (CHIMI), tetrahydropyrimidine-nitromethylene-imidacloprid (THPCHIMI), chloro-thiazolyl-imidacloprid (ClTMNI), thiazolidine-nitromethylene-imidacloprid (SCHIMI), and finally nicotine (NIC) and nicotinoids (+)- and (-)-epibatidine, desnitro-imidacloprid (DNIMI), desnitro-tetrahydropyrimidine-imidacloprid (DNTHP), descyano-thiacloprid (DCNT) and descyano-thiacloprid-olefin (DCNTO).

Table S1. Docking scores and pIC_{50} of neonicotinoids and nicotinoid metabolites. pIC_{50} values are derived from photoaffinity labelling assays of imidacloprid (IMI), desnitro-imidacloprid (DNIMI), thiacloprid (THIAC), descyano-thiacloprid (DCNT) und descyano-thiacloprid-olefin (DCNTO). IC_{50} values were extracted from [28,29] and subsequently converted to pIC_{50}. Docking scores are reported in kcal/mol. Proteins that are used for the docking study are indicated with their PDB-ID; if a water molecule was present in the initial structure, ws1 and ws2 is added to the PDB-ID. PDB-ID-ws2 indicates that the water was removed from the binding site. If there was no water molecule co-crystallised within the binding site, the protein structure is indicated with its PDB-ID alone.

	compound	PDB-ID	docking score	mean docking score	pIC_{50}
**α7**	IMI	7kox-ws2	-3.854	-3.91	3.7
		7koq	-3.972		
	DNIMI	7kox-ws1	-9.479	-10.58	5.0
		7koq	-11.685		
	THIAC	7kox-ws1	-2.591	-1.61	4.0
		7koq	-0.634		
	DCNT	7kox-ws1	-10.879	-10.25	5.2
		7koq	-9.617		
	DCNTO	7kox-ws1	-9.710	-9.88	5.4
		7koq	-10.057		

	compound	PDB-ID	docking score	mean docking score	pIC_{50}
**α3β4**	IMI	6pv7-ws2	-5.281	-4.86	4.9
		6pv8-ws2	-4.430		
	DNIMI	6pv7-ws2	-13.110	-11.76	7.9
		6pv8-ws1	-10.361		
	THIAC	6pv7-ws1	-5.870	-5.69	/
		6pv8-ws2	-5.510		
	DCNT	6pv7-ws2	-11.667	-11.06	/
		6pv8-ws1	-10.449		
	DCNTO	6pv7-ws2	-11.656	-10.20	/
		6pv8-ws1	-8.753		

Table S2. MM-GBSA calculation results using OPLS_2005 versus OPLS4 force fields, respectively.

	compound	PDB-ID	OPLS_2005	mean OPLS_2005	OPLS4	mean OPLS4
$\alpha7$	IMI	7kox-ws2	-24.79	-38.18	-45.53	-45.12
		7koq	-51.56		-44.70	
	DNIMI	7kox-ws1	-49.94	-55.03	-64,60	-60.96
		7koq	-60.11		-57.32	
	THIAC	7kox-ws1	-38.20	-36.51	-52.55	-51.51
		7koq	-34.82		-50.47	
	DCNT	7kox-ws1	-51.40	-57.20	-65.48	-64.57
		7koq	-63.00		-63.66	
	DCNTO	7kox-ws1	-61.48	-65.12	-70.93	-70.42
		7koq	-68.76		-69.90	
	**compound**	**PDB-ID**	**OPLS_2005**	**mean OPLS_2005**	**OPLS4**	**mean OPLS4**
$\alpha3\beta4$	IMI	6pv7-ws2	-27.11	-22.21	-32.63	-31.88
		6pv8-ws2	-17.31		-31.12	
	DNIMI	6pv7-ws2	-47.55	-45.26	-66.63	-65.45
		6pv8-ws1	-42.96		-64.27	
	THIAC	6pv7-ws1	-33.32	-37.15	-39.11	-42.20
		6pv8-ws2	-40.98		-45.29	
	DCNT	6pv7-ws2	-45.46	-47.23	-64.26	-68.27
		6pv8-ws1	-48.99		-72.28	
	DCNTO	6pv7-ws2	-66.44	-64.16	-64.81	-65.80
		6pv8-ws1	-61.87		-66.78	

Representative docking poses

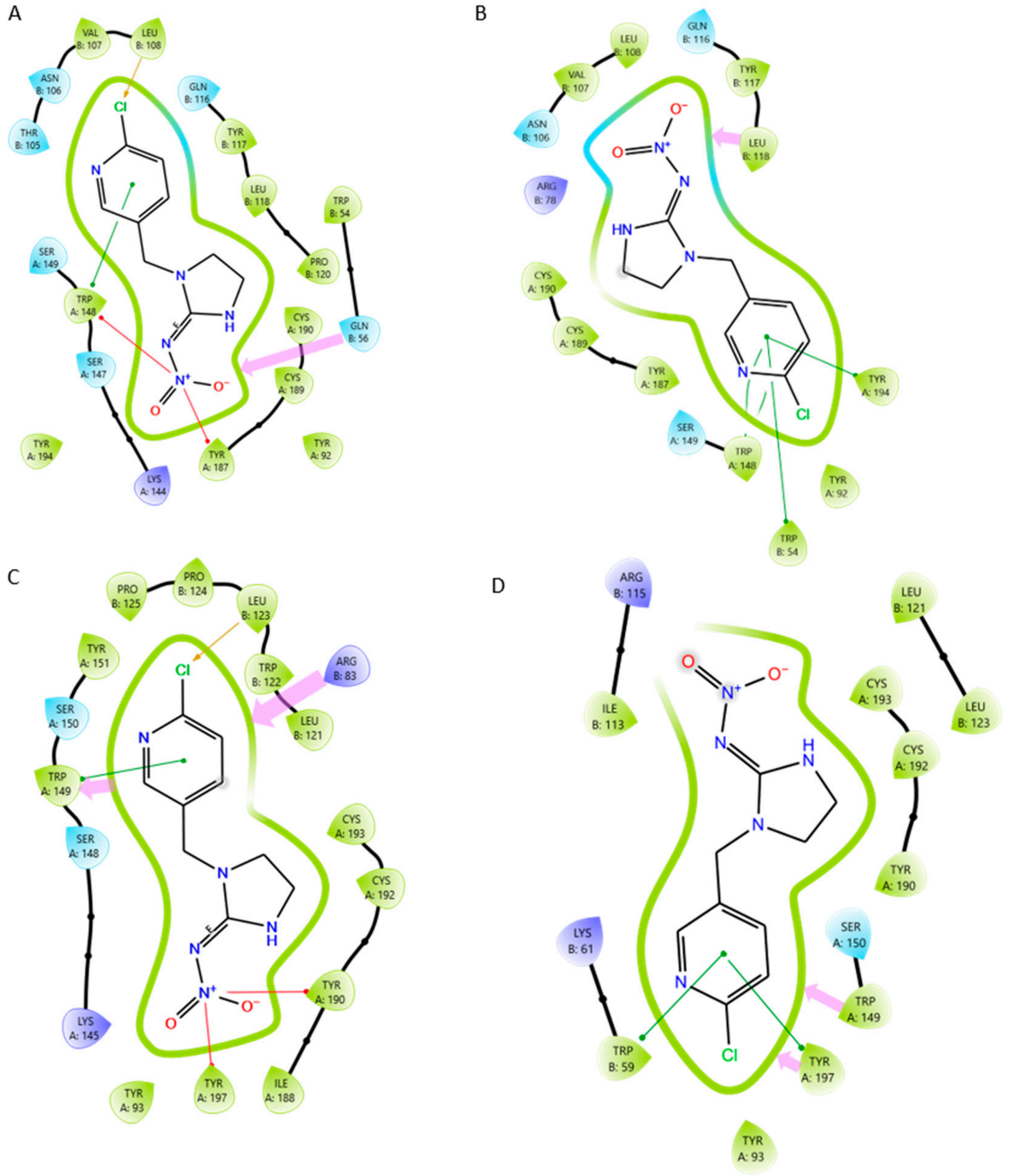

Figure S2. Ligand interaction diagram of representative docking poses of imidacloprid (IMI) in human nAChR $\alpha7$ and $\alpha3\beta4$. Cation-π and π-π-interactions are shown in red and green lines, respectively. Halogen bonding is indicated by orange arrows. Hydrogen bonds that are maintained during the MD simulation are shown in wide purple arrows; (**A**) IMI in common binding mode in 7kox-ws2; IMI serves as hydrogen bond acceptor for Gln56 for 26.55% of the 50ns MD simulation; (**B**) IMI in inverted binding mode in 7koq; IMI serves as hydrogen bond acceptor for the side chain of Leu118 for 67.86% of the simulation time; (**C**) IMI in common binding mode in 6pv8-ws2; IMI serves as hydrogen bond acceptor for Arg83 for 10.58% of the MD simulation time, additionally a Trp149 is the hydrogen bond acceptor for 16.37% of the MD trajectory; (**D**) IMI in inverted binding mode of 6pv7-ws2; Hydrogen bonds are formed from Tyr197 and Trp149 to IMI during 14.37% and 13.77% of the 50ns-MD-trajectory, respectively.

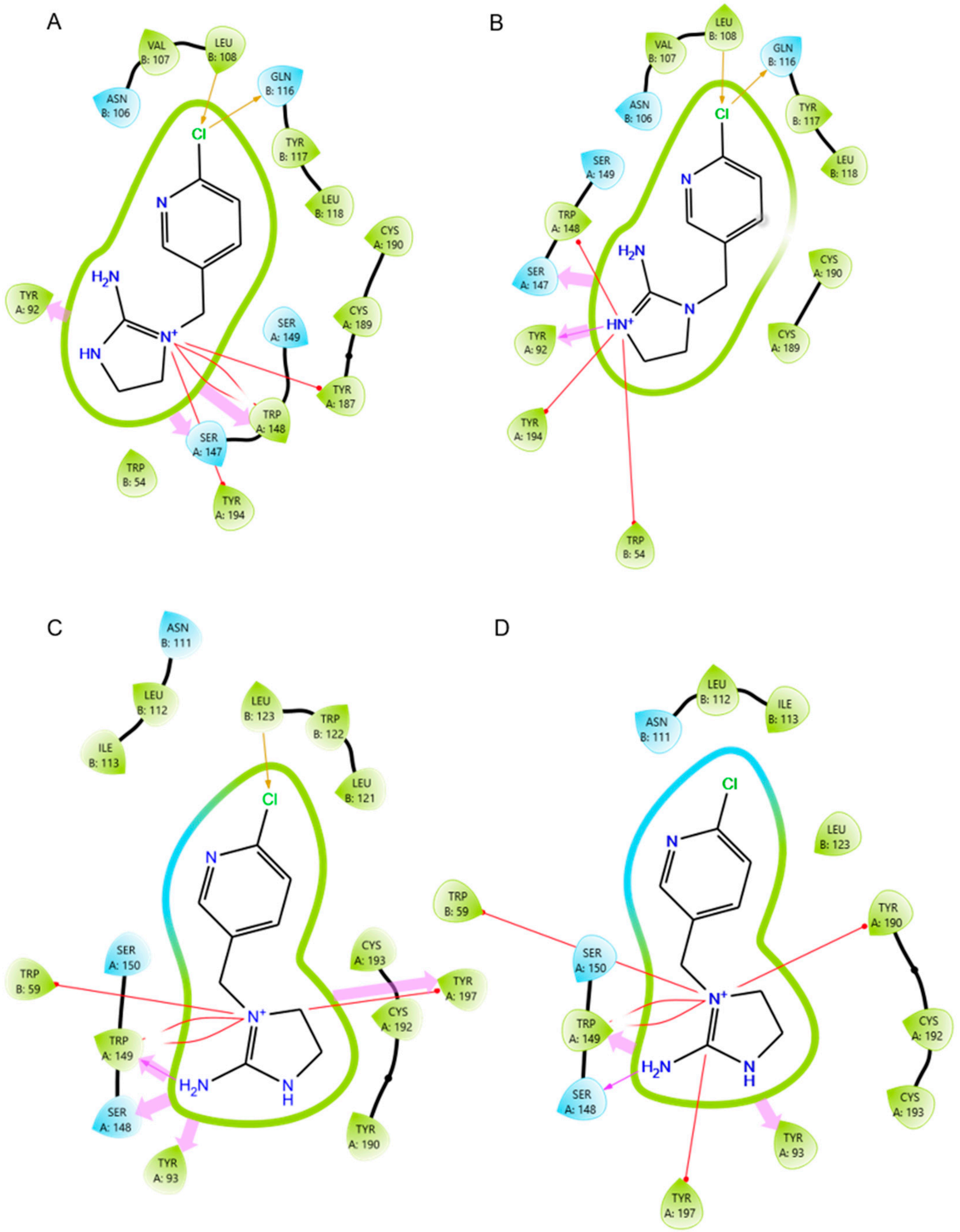

Figure S3. Ligand interaction diagram of representative docking poses of desnitro-imidacloprid (DNIMI) in human nAChR $\alpha 7$ and $\alpha 3\beta 4$. Cation-π interactions are shown in red lines. Halogen bonding and hydrogen bonding is indicated by orange and purple arrows, respectively. Hydrogen bonds that are maintained during the MD simulation are shown in wide purple arrows. (**A**) DNIMI in 7kox-ws1 ($\alpha 7$); three main hydrogen bonds are formed during the 50ns simulation time: Tyr92 is a hydrogen bond acceptor for 99.80% of the MD trajectory. Additional hydrogen bonds are formed to Ser147 and Trp148, that are maintained for 25.16% and 11.98%, respectively; (**B**) DNIMI in 7koq ($\alpha 7$): two hydrogen bonds are formed to the main chain of Ser147 and to the side chain of Tyr92 for 70.66% and 74.05%, respectively; (**C**) DNIMI in 6pv7-ws2 ($\alpha 3\beta 4$); 4 residues maintain main hydrogen bonds during the MD simulation: main chain of Trp149 for 97.92%, and side chains of Ser148 for 22.16%, Tyr93 for 60.48%, and Tyr197 for 14.57%; (**D**) DNIMI in 6pv8-ws1 ($\alpha 3\beta 4$); DNIMI is a hydrogen bond donor for the main chain of Trp149 for 60.66% and to the side chain for 22.75% of the MD trajectory. The hydrogen bond to Tyr93 was maintained for 46.71% of the 50ns simulation.

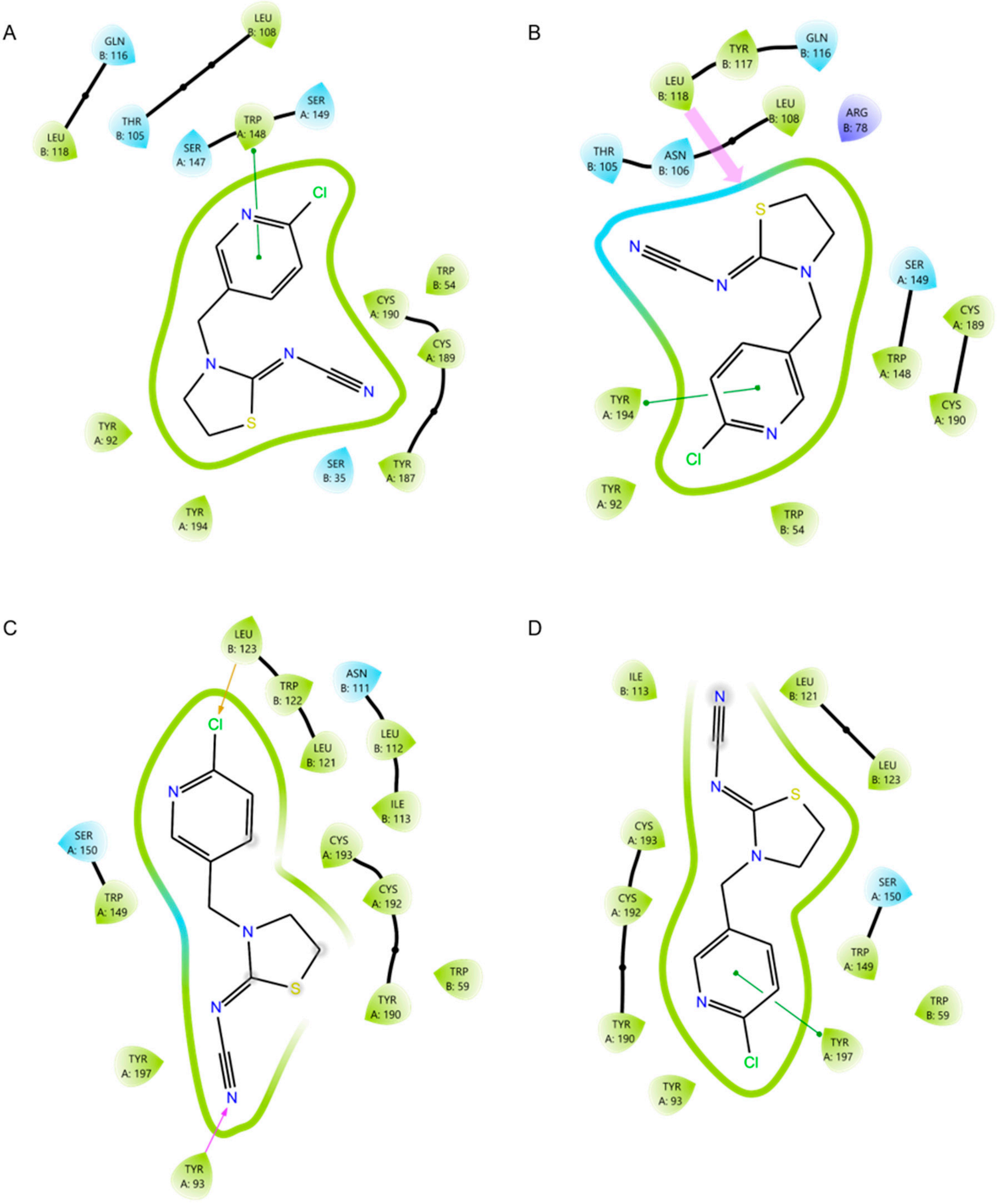

Figure S4: Ligand interaction diagram of representative docking poses of thiacloprid (THIAC) in human nAChR α7 and α3β4. π-π-interactions are shown in green lines. Hydrogen bonding is indicated by purple arrows. Hydrogen bonds that are maintained during the MD simulation are shown in wide purple arrows. (**A**): THIAC in its common binding mode in 7kox-ws1 (α7); (**B**) THIAC in inverted binding mode in 7koq (α7); a hydrogen bond from Leu118 was maintained for 51.1% of the MD simulation; (**C**) THIAC in common binding mode in 6pv7-ws1 (α3β4); (**D**) THIAC in inverted binding mode in 6pv8-ws2 (α3β4).

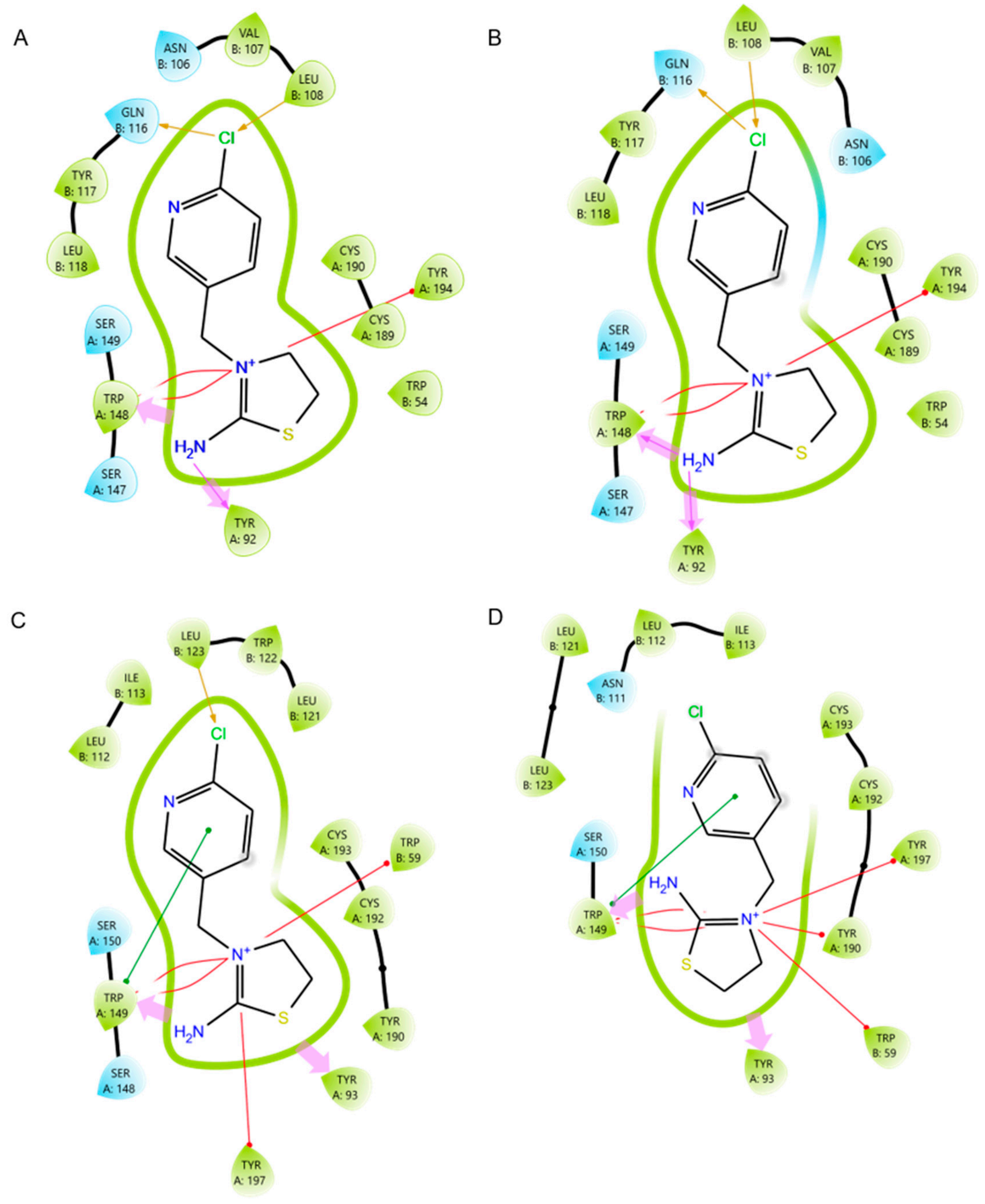

Figure S5: Ligand interaction diagram of representative docking poses of descyano-thiacloprid (DCNT) in human nAChR α7 and α3β4. Cation-π and π-π-interactions are shown in red and green lines, respectively. Halogen bonding and hydrogen bonding is indicated by orange and purple arrows, respectively. Hydrogen bonds that are maintained during the MD simulation are shown in wide purple arrows; (**A**) DCNT in 7kox-ws1 (α7). The main and side chain of Trp148 serve as hydrogen bond acceptor for 51.30% and 17.56%, respectively. Tyr92's side chain maintains a hydrogen bond for 34.33% of the simulation time; (**B**) DCNT in 7koq (α7); For 63.87% of the MD trajectory, a hydrogen bond is formed to the main chain of Trp148. Tyr92 serves as hydrogen bond acceptor for 49.90% of the 50ns MD simulation. Both of these hydrogen bonds are also formed in the representative docking pose, which is indicated in thin purple arrows; (**C**) DCNT in 6pv7-ws2 (α3β4); Trp149 and Tyr93 are hydrogen bond acceptors for 55.09% and 38.32% of the MD trajectory, respectively; (**D**) DCNT in 6pv8-ws1 (α3β4); Trp149 and Tyr93 are hydrogen bond acceptors for 73.05% and 55.69% of the MD trajectory, respectively.

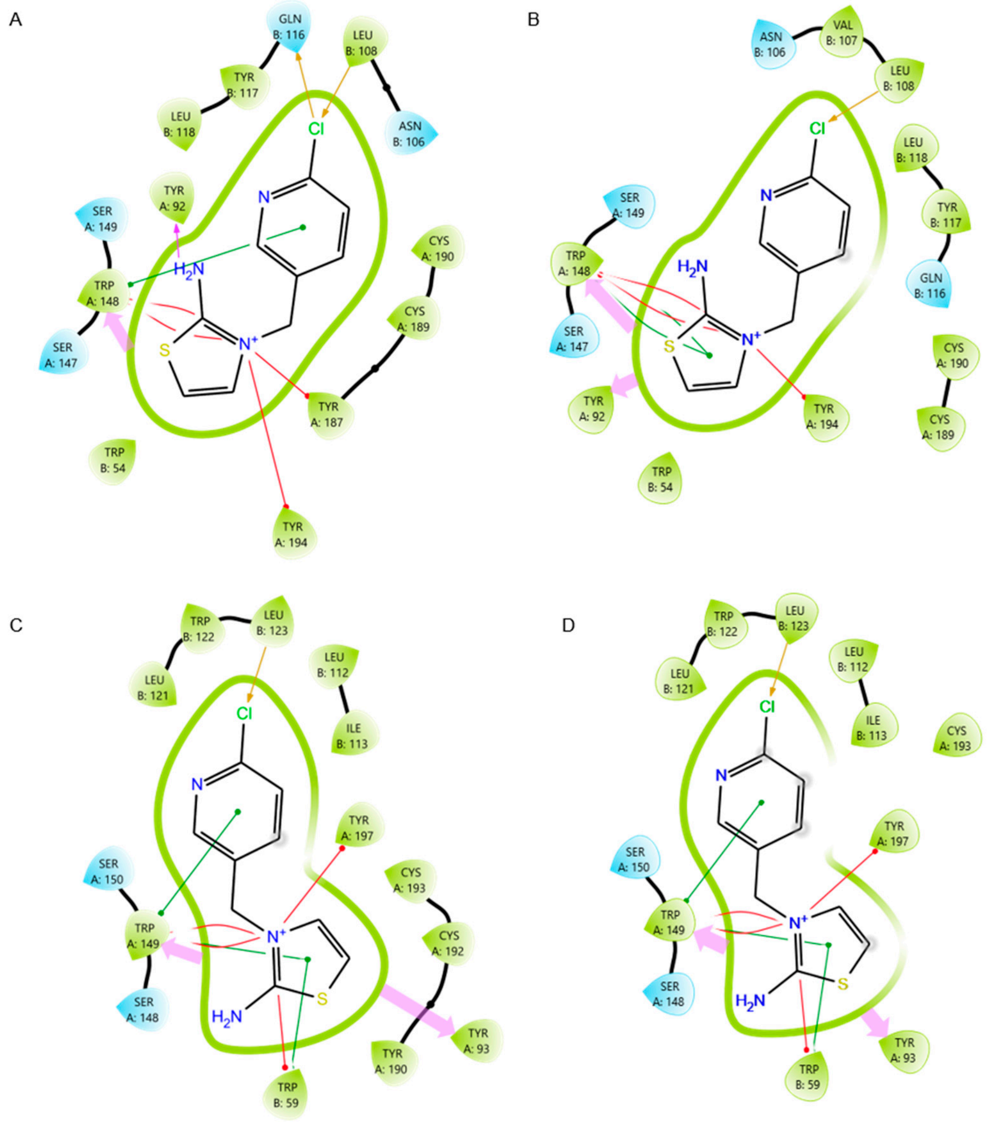

Figure S6: Ligand interaction diagram of representative docking poses of the olefin derivative of descyano-thiacloprid (DCNTO) in human nAChR α7 and $\alpha3\beta4$. Cation-π and π-π-interactions are shown in red and green lines, respectively. Halogen bonding and hydrogen bonding is indicated by orange and purple arrows, respectively. Hydrogen bonds that are maintained during the MD simulation are shown in wide purple arrows; (**A**) DCNTO in 7kox-ws1 (α7); The main and side chain of Trp148 serve as hydrogen bond acceptor for 70,66% and 38.92% of the 50ns simulation, respectively; (**B**) DCNTO in 7koq (α7); The main and side chain of Trp148 serve as hydrogen bond acceptor for 70.66% and 13.97% of the 50ns simulation, respectively. In this complex an additional hydrogen bond to Tyr92 is formed for 34.33% of the MD trajectory; (**C**) DCNTO in 6pv7-ws2 ($\alpha3\beta4$); The main chain of Trp149 is a hydrogen bond acceptor for 54.49% of the MD simulation. For 66.47% of the MD simulation, a hydrogen bond is formed to Tyr93; (**D**) DCNTO in 6pv8-ws1 ($\alpha3\beta4$); The main and side chain of Trp149 serve as hydrogen bond acceptor for 63.67% and 15.57% of the 50ns simulation, respectively. Tyr93 forms a hydrogen bond for 28.74% bond of the simulation time.

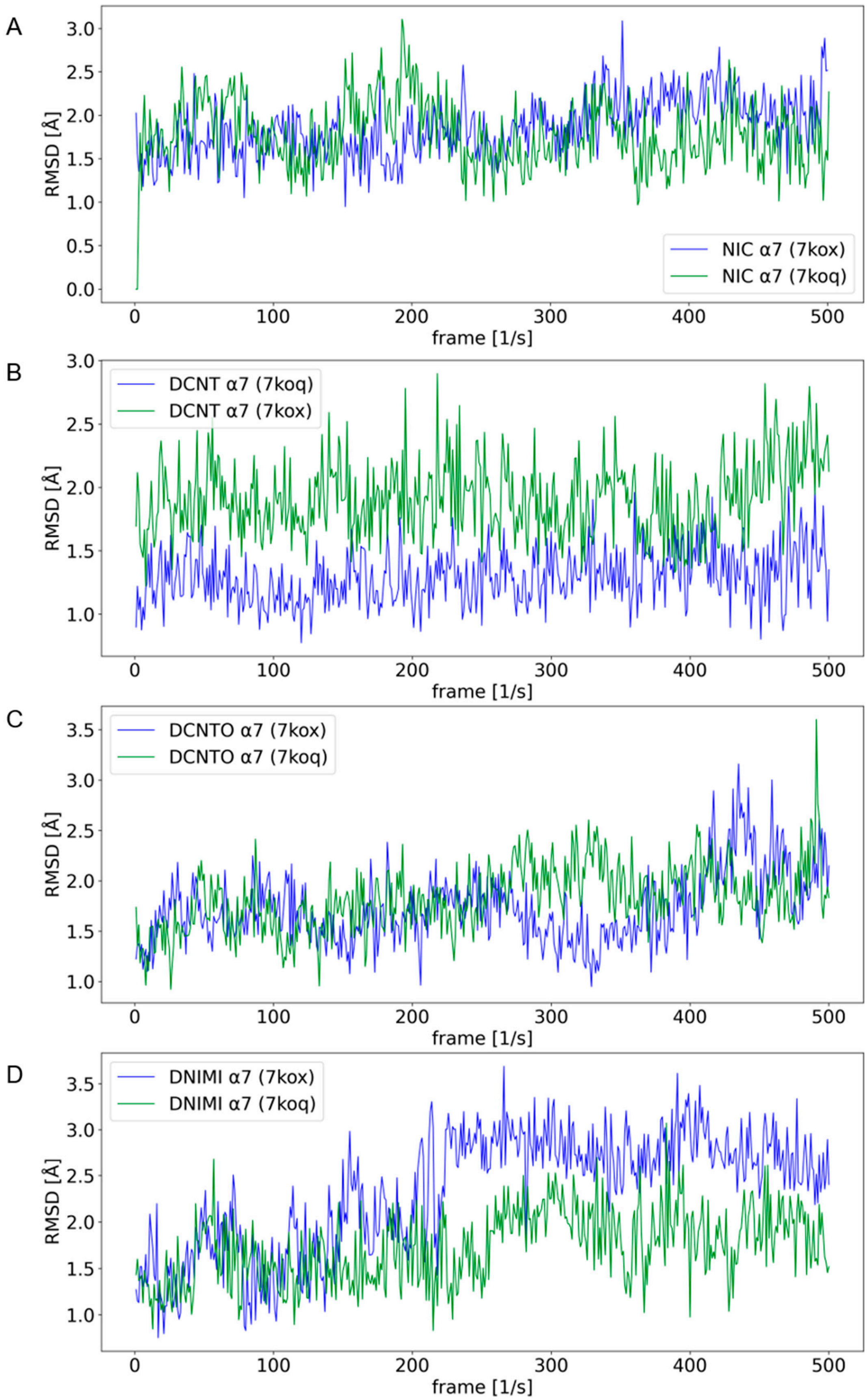
A
B
C
D
RMSD [Å]
frame [1/s]
NIC α7 (7kox)
NIC α7 (7koq)
DCNT α7 (7koq)
DCNT α7 (7kox)
DCNTO α7 (7kox)
DCNTO α7 (7koq)
DNIMI α7 (7kox)
DNIMI α7 (7koq)

Figure S7. 1D-RMSD plots of the compounds from two different starting conformations that were subjected to the 50ns MD simulation in the α7 isoform: (**A**) nicotine (NIC), (**B**) descyano-thiacloprid (DCNT), (**C**) descyano-thiacloprid-olefin (DCNTO), and (**D**) desnitro-imidacloprid (DNIMI).

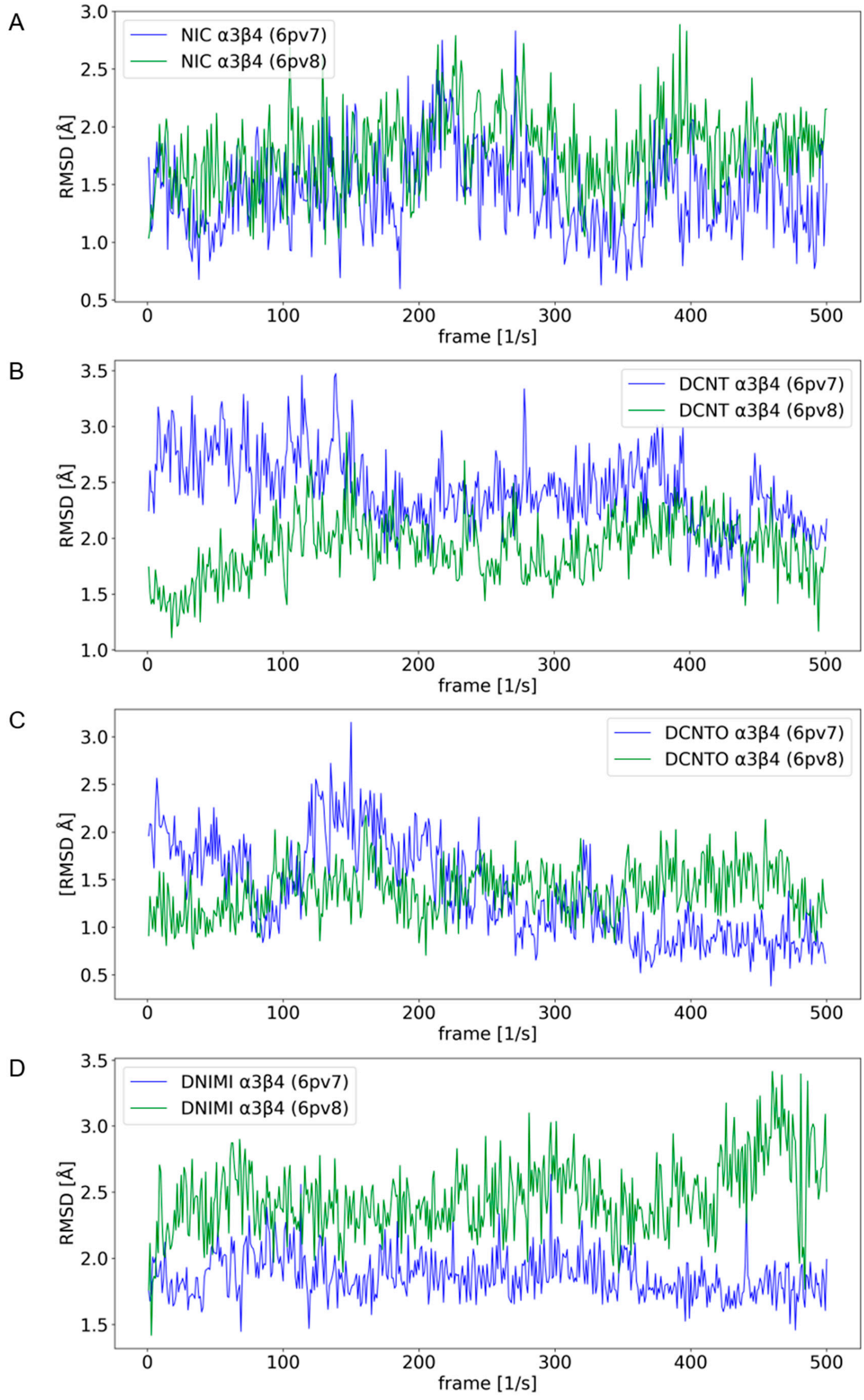

Figure S8: 1D-RMSD-plot of the compounds from two different starting conformations that were subjected to the 50ns MD simulation in nAChR *α*3β4: (**A**) nicotine (NIC), (**B**) descyano-thiacloprid (DCNT), (**C**) descyano-thiacloprid-olefin (DCNTO), and (**D**) desnitro-imidacloprid (DNIMI).

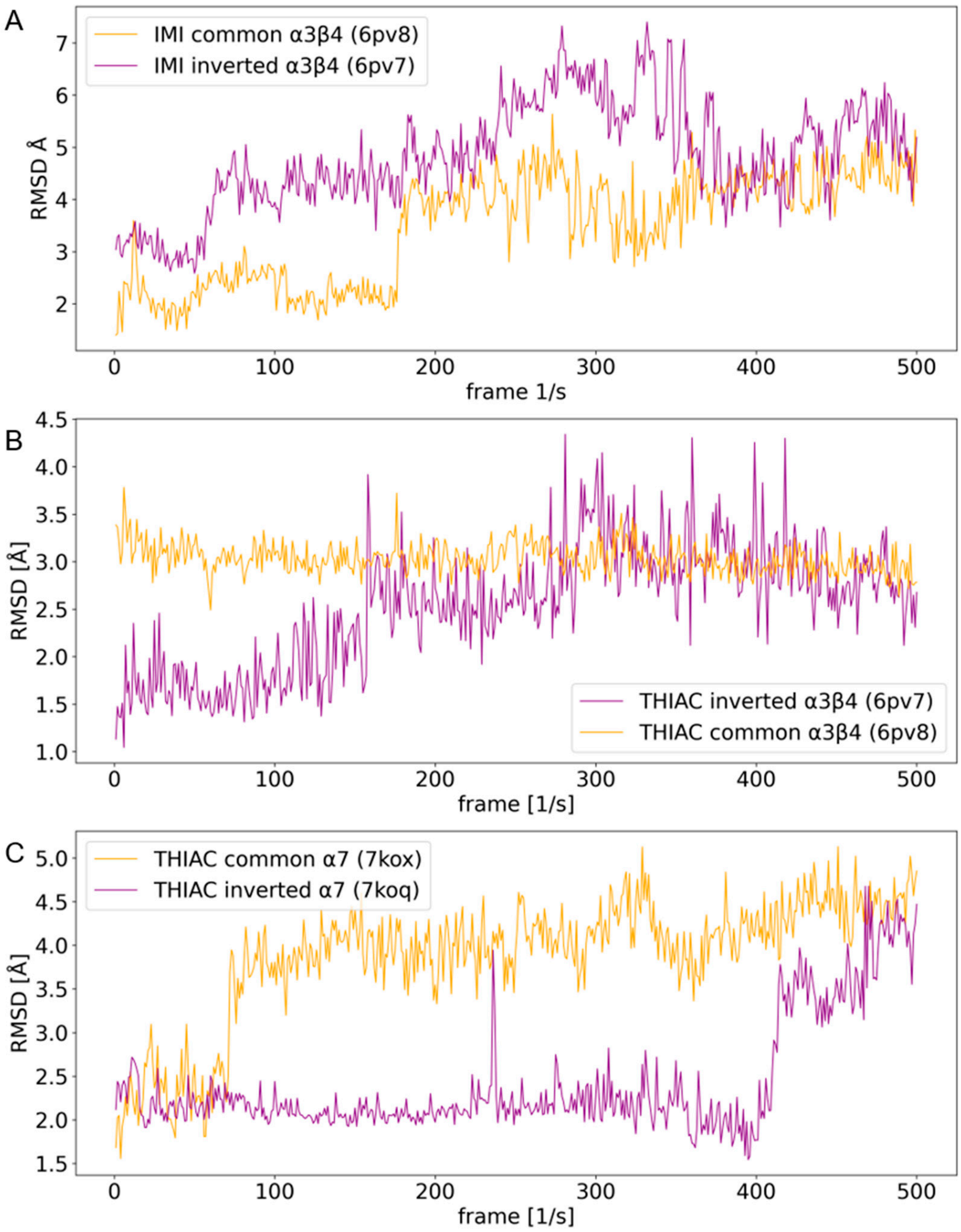

Figure S9. 1D-RMSD plots of the compounds from two different starting conformations that were subjected to the 50ns MD simulation: (**A**) imidacloprid (IMI) in $\alpha3\beta4$; (**B**) thiacloprid (THIAC) in $\alpha3\beta4$; (**C**) thiacloprid (THIAC) in $\alpha7$. The 1D-RMSD plot of IMI in $\alpha7$ is shown in the main text in figure 3C.
